# "The Hospital" Nursing Mirror

**Published:** 1901-06-01

**Authors:** 


"The Hospital" Nursing Mirror.

Being the Nursing Section of "The Hospital."

[Contributions for this Section of "The Hospital" should be addressed to the Editor, "The Hospital" Nursing Mirror, 28 & 29 Southampton Street, Strand, London, W.C.]

### Notes on News from the Nursing World.

### THE WOMEN'S MEMORIAL TO QUEEN VICTORIA.

It is satisfactory to learn that the movement initiated at Londonderry House a few weeks ago for the permanent endowment of Queen Victoria Jubilee Institute for Nurses is making progress in the country. At a committee meeting last week, which was attended by a number of members, it was reported that organisations were already at work in twenty-two counties and a large number of towns, that energetic steps were being taken to put the appeal before every woman in the United Kingdom, and that many women's organisations in all classes of society were taking part. Moreover, as signified in our columns last week, the Countess of Cadogan has succeeded in enlisting influential support for the Women's Memorial to Queen Victoria in Ireland. Persistence as well as energy will, however, have to be maintained in order to ensure success, and while it is a great advantage that ladies of high rank and social position should be at the head of the movement, the importance of all classes of women joining in it cannot be too strongly emphasised.

### DEATH OF AN ARMY NURSING SISTER.

We much regret to learn that the protracted illness of Nursing Sister Edwards from enteric fever terminated fatally at Bloemfontein last Wednesday. Miss Gertrude Stockley Edwards, who entered the Army Nursing Service Reserve last August, was very little over thirty when she fell a victim to the disease which has done such deadly mischief in South Africa. She was trained at the Sheffield Royal Hospital, and after doing private nursing in Bath and London, returned to the same institution as staff nurse. In November, 1899, she became charge nurse at the Grove Fever Hospital, an appointment she relinquished on her admission to the Army Reserve.

### IRELAND AND THE NURSING QUESTION.

There is some reason to hope that the nursing question in Irish poor law infirmaries will not be made the shuttle-cock of political parties. Even Irish papers which have not concealed their dislike to the intervention of the Local Government Board, admit that the revelations made in the course of the proceedings before the Judicial Committee of the Privy Council, which were reported at length in our columns last week, are "simply shocking." Thus, the *Irish Independent*, while characterising the claim advanced by the Board as a preposterous one, admits that "no one will be likely to argue that all our workhouse hospitals are now in an absolutely perfect condition, and may safely be left without exterior supervision of any kind;" and the *Freeman's Journal* says, "it cannot be denied that in the motive alleged for the nursing order, and in the case put forward to support it, the Board should command general public sympathy." From the *Freeman's Journal* we also take the following:—

The level of intellect obtaining among some poor law administrators is illustrated by the objection which was made not long ago at a public meeting to a proposal for the appointment of a night nurse. The infirmary was quite destitute of care during the night except for what the inmates could give each other. "It's all nonsense," said the modern Bumble stoutly. "Sick people ought to be left to go to sleep at night, not waked up to be nursed!"

We rejoice that our Nationalist contemporary supports our suggestion that a new order should at once be made dealing with the question of assistant nurses, and expresses its conviction that there is "no popular constituency" throughout the unions of Ireland that "would elect a poor law guardian who for the sake of £8 a year would inflict unnecessary suffering on a single inmate of a union hospital or a lunatic ward." But this is in curious contrast with the statement of Dr. Smith, medical officer of Naas Union, who told the Judicial Committee of the Privy Council that "nursing in the modern sense was not appreciated by local boards"—a statement which it has been abundantly proved applies both to Ulster and to the South. It is not a case of the Armagh Workhouse Infirmary, where there are two trained nurses and one temporary nurse for 48 patients and 43 lunatics, nor of the South Dublin Workhouse, where there is but one trained nurse at night for upwards of 1,500 patients. We are satisfied that the whole, or nearly the whole, of the boards of guardians in Ireland are tarred with the same brush, and we fear that the task of compelling them to do their duty to the sick and suffering poor will tax the resources if not exhaust the patience of the executive.

### "A DELICATE SUBJECT."

Our correspondent, "A Plague Nurse," who writes to us from Capetown, rightly says that she touches on a subject of extreme delicacy. But it is also one of very great moment. In effect, she charges some of the nurses who have gone out to South Africa under the auspices of the Colonial Office with unseemly behaviour during the voyage. It would be unreasonable to suppose that all nurses, even the most capable, should be patterns of discretion, and we are not therefore able to blame the authorities who made the selection for plague duty if some of their nominees have violated the canons of good taste. At the same time it is a matter for deep regret when the dignity of the calling is not maintained by those who should be most concerned to uphold it. Prevention, however, is better than cure, and when a number of nurses who are young enough to be thoughtless are sent across the seas, and hourly thrown into the society of men with nothing to do, it would be an obvious safeguard if one of them, to whom added years have brought added experience, were charged with the obligations and authority of a matron.

### MORE NURSES' HOMES.

There is no doubt that of local memorials to Queen Victoria one of the most popular is the erection of a Nurses' Home. We have already mentioned several instances, and among other places in which it has been decided to adopt this mode of doing honour to the late Sovereign are Rochdale, Hereford, and Chatham. At Rochdale, the need of a home will soon be practically felt, as, although the work of the district nurses is steadily increasing, and the number of nurses will certainly have to be augmented, the house at present occupied by the staff can only be made to accommodate one more. At Hereford it has been enthusiastically determined to try and raise a sum of £1,500 for the purpose; and the alternative schemes which were suggested at a meeting in the Guildhall met with little support. There was no note of dissent raised at Chatham when the project was brought forward by the Mayor, and several handsome contributions were announced at once. The house in which the district nurses at Chatham at present reside is not suitable. It is hoped that the start will have been made and the foundation-stone of the new home laid before the annual meeting of the Nursing Association takes place next year.

### NURSES IN YET ANOTHER NEW LIGHT.

It came out clearly at the annual meeting of the West Hartlepool District Nursing Association that more nurses are badly needed, and that the position of the association would justify an immediate, if modest, augmentation of the staff. Last year the two nurses paid 7,406 visits to 261 patients, an increase of 1,314 over the previous year; and there is a balance in hand of £313. In these circumstances there does not seem to be any necessity for hesitation. That the miners of Hartlepool appreciate the work of the nurses was shown in a speech by the Rev. W. J. Knowlden, who moved the adoption of the report. He said that "he knew of one case where a nurse was brought into the country from one of the nearest towns, and where there was an epidemic of typhoid, and her presence at once made one feel quite confident that everything would be looked after. One of the miners was led to make the remark, 'I think nurses is one of the best things that has come out.' So they were regarded as a sort of patent." We do not suppose that any nurse objects to be considered "a sort of patent," so long as her patients are willing and glad to do as she tells them.

### A SELFISH SISTER.

There is an old story of a village clergyman who delivered excellent sermons, but who, conscious of the fact that his practices were open to criticism, used to say to his hearers, "Do as I say, and not as I do." He was, at any rate, candid; but the people who preach good habits and practise bad ones are apt, in some circumstances, to excite irritation. "Tired Night Nurse," who states in another column that she is on duty in a small hospital from 10 p.m. till 9 a.m., naturally feels indignant with the sister who not only enforces punctuality upon the nurses, but herself invariably arrives an hour, and sometimes nearly an hour and a half, after 9 a.m., when she is due. Her inconsistency is sufficiently glaring, but her selfish disregard of the health of the nurses, who cannot leave the hospital until she arrives, cannot be too severely condemned.

### SECTARIANISM AT CORK.

It is understood that the decision to appoint a member of a religious order as matron of the Cork South Charitable Infirmary and County Hospital will result in the substitution throughout of nuns for the trained nurses who have hitherto formed the staff. The action of the majority of the committee is the more superfluous because the minority offered no objections to the appointment of a lay matron belonging to the Roman Catholic Church. As Mr. Newsom, whose speech was admirable in tone and in temper, put it, "there are plenty of Roman Catholic nurses who are well trained and who are perfectly capable of acting as matron of the institution, and who would do full justice to the trust reposed in them." Another objection to the new departure is that, while the Romanist subscriptions to the Infirmary amount to less than £100, the Protestants contribute upwards of £400. Moreover, as Mr. Newsom also pointed out, the probationers, whose money the committee have taken and who are with them at present, did not expect to have a lady in religious orders put over them. In fact, the change has evidently been determined upon as a sectarian movement, and as such it cannot be too much deplored. It is the more unjustifiable because even its advocates were not able to allege that the nursing at the infirmary under the *régime* which is now to be swept away, had not been of a satisfactory character.

### THE HOLIDAY QUESTION AT BRISTOL.

We regret to learn that, in spite of the decision of the Bristol Board of Guardians to increase the number of probationers by two, they have declined to sanction the proposal to extend the holidays of the nurses at the workhouse infirmary. A resolution to the effect that the holidays of the charge nurses should be fixed at three weeks and two days, and of the assistant nurses and second and third year probationers at three weeks, instead of a fortnight, though strongly supported, especially by Miss Woollam, who said that the nurses required more than a fortnight's holiday, and that several left last year because of the short holiday granted, was lost by two votes —27 voting for, and 29 against. This is how, according to a local paper, one of the guardians named Bastow thought proper to treat the matter:—

> Two things he did not favour—bicycles in the streets ridden by ladies, and nurses walking about the footpaths. If they gave nurses three weeks' holiday, they would walk about more than they did. They wanted more air space and less nurses, not one at the foot, one at the head, and one each side of the patient. If the nurses had three weeks' holiday, they would for three months talk about where they went, what they heard, and what they saw.

Another of the opponents of the extension of time urged that "whenever a nurse required extra holiday through ill-health the Board granted it." But one way to keep the nurses in good health is to allow them a sufficiently long holiday, and a "holiday" rendered compulsory by ill-health is not an event to anticipate with pleasure. We expect that, as the majority of the Bristol Guardians were warned, the ungracious policy determined upon will increase the difficulty of the Board in obtaining the most desirable nurses.

### PROPOSED TOWN'S NURSE FOR SWINDON.

The memory of Queen Victoria is probably to be commemorated in Swindon by raising a fund to assist the Victoria Hospital to provide a town's nurse. The question has been raised at two public meetings, and the decision arrived at on the second occasion was that the joint committee, consisting of the Victoria Hospital Committee and a committee of twenty outsiders, should invest the amount subscribed at a collection on May 24, and "if practicable" devote the income to the provision of a town's nurse. Several speakers pointed out that one was badly needed, and the facilities offered at the Victoria Hospital, where bandages and dressings can be had in case of emergency, were urged in favour of the movement. It had previously been proposed to establish a Victoria Scholarship in connection with the Swindon and North Wilts Technical School, or to found an art gallery, but these proposals have been finally abandoned, and the feeling in Swindon is apparently strongly in favour of the nursing scheme, which as a speaker at the meeting said, is one that everybody without distinction of class or creed could support.

### HOSPITAL NURSES AND TENNIS.

At a meeting of the Huddersfield Borough Council, the question of a tennis court at the Sanatorium was discussed. Alderman Woodhead said it was a needless waste of money to provide a tennis court for a hospital, "for if the patients were sufficiently recovered to be able to play tennis they ought to be discharged." But it was at once pointed out that the tennis court was not intended for the patients so much as for the nurses of the institution; and Councillor Aston declared that "it was the least the Council could do to endeavour to provide the nurses with some means of healthy recreation during the time they are off duty." This sensible view apparently commended itself to the other councillors, as no further objection was raised.

### ROYAL BRITISH NURSES' ASSOCIATION.

The annual meeting of the Royal British Nurses' Association will be held on Thursday, June 6th, at the Imperial Institute, South Kensington, commencing at 3 P.M. The chair will be taken by Sir James Crichton-Browne. At the conclusion of the meeting a reunion will be held at the Crystal Palace, where arrangements have been made for members to have tea together, and travel back by special train. Tickets and all further particulars can be obtained from the Secretary, 10 Orchard Street, W.

### IPSWICH NURSES AND TYPHOID.

In the annual report on the sanitary condition of the borough and port of Ipswich, by Dr. Elliston, medical officer of health, allusion is made to the re-organisation of the nursing staff at the Borough Fever Hospital. The matron was able to complete the nursing staff before the pressure of typhoid fever which took place in October. There are now four charge nurses, and eight probationers, and the medical officer says that the thanks of the sanitary authorities are due to the matron and the nursing staff "for the highly efficient manner in which they have carried out their arduous and dangerous duties. "Unfortunately," he adds, "three nurses or one fourth of the whole staff contracted typhoid in the discharge of their duties; they were exceptionally long and severe cases, and although they happily escaped with their lives, the health of two was so shattered that they had to give up nursing as an occupation." This is an unusual, as well as a distressing, experience.

### PROGRESS AT REDDITCH.

The annual meeting of the Redditch and District Nursing Association was of an encouraging character. The chairman read the report, which showed that 12,187 visits had been paid in 1900, an increase of 426 over the previous year; that an inspector from Queen Victoria's Jubilee Institute had twice visited Redditch during the twelve months, and that on both occasions the result was pronounced satisfactory; that a concert organised in September produced the substantial sum of £33; and that the income of the association had exceeded the expenditure by nearly £24. A suggestion was again made by Mr. Bartlet that the report and balance-sheet of the association, together with any alteration of rules which might be proposed, should be sent to each subscriber with the notice convening the meeting. "If that were done," he urged, "the members would have a better chance of digesting them than they now had," from which we infer that the subscribers do not, under existing conditions, get the documents named when they receive the notice of the meeting. The suggestion seems to have been ignored, but we see no reason why it should not be adopted.

### THE ASYLUM WORKERS.

In some respects the fourth annual report and financial statement of the Association of Asylum Workers, whose president's speech at the meeting will be found in another column, is not satisfactory. The aggregate receipts were £237, as compared with £255 in 1899, and the number of new members elected was 963, as against 1,010. On the other hand, it is encouraging to find that an increasing interest is taken by workers in the Colonies to bring the Association within the reach of all who are engaged in the care of the insane, in every part of the King's dominions. The fact that eleven cases were sent to approved homes at health resorts by means of the "Home of Rest" Fund, for periods of three weeks or more, attests its value. The remarks of Sir James Crichton-Browne on the strain to which asylum workers are subjected, and the sympathy they merit, were very much to the point.

### CARLISLE AND DISTRICT ASSOCIATION

The annual report of the Carlisle District Nursing Association, which was adopted at the meeting, shows that the number of patients was 411, and the number of visits paid 11,437. The Association has now been established ten years, and the districts of the three nurses employed embrace the whole of the city. A return accompanying the report gives the class of people among whom the services of the nurses were in request, the principal being as follows:—Labourers, 81; railwaymen, 41; millhands, 42; small shops and assistants, 30; mechanics, 27; parish patients, 36; lodging-house keepers, 26; soldiers, 23. Dr. Barnes, who proposed a vote of thanks to the honorary secretary, Mrs. Scott Steele, mentioned that no nurse who had not received three years' hospital training was accepted, and no one was allowed to act as nurse except under medical supervision. The committee very wisely adhere to these conditions, and find the result extremely satisfactory. Hitherto the nurses have been provided with free passes on the trams, but there are apprehensions that this unusual privilege will shortly be discontinued.

### THE PROBATIONER AND THE COSTERMONGER.

A young probationer in one of the male wards of a large London hospital was much amused by a costermonger's apparent admiration of herself, and her work as a nurse. In a confidential moment he remarked with charming candour, "I reckon, nurse, you an' me wiv a barrer atween us could do a roarin' tride."

### SHORT ITEMS.

The jury at the inquest on the bodies of the persons who lost their lives by the fire at Stafford Workhouse added a rider to their verdict recognising the services of the nurse who was so largely instrumental in arresting the spread of the conflagration.—The annual meeting of the Friedenheim Hospital (House of Peace for the Dying) will be held on Thursday next at 3.30 in the Lecture Hall of the School for the Blind, 10 Upper Avenue Road, Swiss Cottage.—In addition to the intimation that Sister Richardson's condition has undergone no change for the better, the latest progress report states that the condition of Sister Culverwell is grave.

### Notes of Surgical Lectures to Nurses.

Delivered at the London Temperance Hospital by Dr. W. J. COLLINS, M.S., B.Sc., D.P.H.Lond., Fellow of London University and of the Royal College of Surgeons. Surgeon to the London Temperance and the Royal Eye Hospitals.

### CIRCULATION AND RESPIRATION.

THE heart, the chief agent in the circulation, is a muscular pump, consisting of two distinct halves and four chambers (two auricles, two ventricles) and weighs 10 ounces. The right side of the heart receives venous blood from the body (by the two great *venæ cavæ*) into the right auricle, and by its ventricle sends it to the lungs to be oxygenated. Return of blood during the ventricular contraction is prevented by the closure of the tricuspid valve which guards the orifice between the right auricle and ventricle; as the semilunar valves (situated at the point where the pulmonary artery leaves the right ventricle) are at the same moment open, the discharge of the contained blood to the lungs is secured, and its regurgitation thence is precluded by the immediately subsequent closure of these pulmonary valves. As the right heart is pulmonic so the left heart is systemic, and the left ventricle is accordingly thrice the thickness of the right in its muscular wall. The oxygenated blood collected into the left auricle from the lungs by the four pulmonary veins pours into the left ventricle, whose subsequent contraction, at the same time as the right, drives the blood into the systemic arteries through the aorta; return to the auricle is precluded by the closure of the mitral valve (analogous to the tricuspid on the right side), the mouth of the aorta being guarded like that of the pulmonary artery by semilunar valves which shut the door when the blood has been expelled from the ventricle.

The pulse is defined as that temporary additional distension which the arteries undergo at each contraction (systole) of the ventricle. A tracing of the pulse wave can be taken by means of the sphygmograph. It varies in rate (60-150 a minute), volume, length, regularity, and compressibility. Blood pressure (equal to from 5 to 8 inches of mercury) is due to the pump of the heart, the elasticity of the arteries and the resistance of the capillaries to the progress of the blood stream. The circulation is complete in half a minute, but the rate at which the blood travels is 1 inch a minute in the capillaries, a foot a second in the arteries, and about half a foot a second in the veins. The beat of the heart is automatic (a frog's heart continues beating though removed from the body), but it is held in check by certain nerves proceeding from the brain (pneumogastric); its contractions and the tone of the arteries are also affected by certain branches of the sympathetic nerve (accelerator, vaso-motor).

The first sound of the heart (lubb) is caused by the closure of the valves between the auricles and ventricles (tricuspid, mitral) at the time of the contraction of the latter (systole); the second sound (dup), heard best over the base of the heart, is caused by the closure of the arterial valves (pulmonary and aortic semilunar) during the pause (diastole) immediately succeeding the systole. The following table shows the relation between the states of the valves and the sounds and murmurs of the heart:—

| | | SYSTOLE. | DIASTOLE. |
|---|---|---|---|
| VALVES | Cuspid | Shut | Open |
| | Semilunar | Open | Shut |
| SOUNDS | ... | First | Second |
| MURMURS... | ... | Systolic | Diastolic |

Murmurs are generally due to valves (especially those of the left side) being rendered incompetent or obstructive as the result of inflammation of the endocardium that lines the heart, as is often the case in acute rheumatism. Similarly the fibro-serous tunic or pericardium which covers the heart may be inflamed (pericarditis) causing a friction sound to be heard on auscultation, or effusion of fluid into its cavity may occur, increasing the area of cardiac dulness on percussion, together with pain, fever, and constitutional distress. The pain and distress which accompany diseases of the heart are often characterised by special agonising symptoms (angina pectoris). Fainting (syncope), dropsy (ascites, anasarca), bleeding from the lungs (hæmoptysis), degeneration of the liver (cirrhosis) are often associated with diseases of the heart.

Aneurism is a tumour containing blood communicating with an artery; it may undergo spontaneous cure by "thrombosis" (coagulation of the blood in the vessels during life), or may be cured artificially by ligature of the artery above the seat of the aneurism.

Respiration is the process whereby oxygen from the air is conveyed by the red corpuscles of the blood to the cells which compose the body. All animals require oxygen, absorbed either directly or through gills from solution in water, or by lungs from air, to carry on combustion which produces heat, carbonic acid and water.

The right heart pumps venous blood to the lungs in the capillaries of which it is freely exposed to the air sucked into the lungs by expansion of the chest which is effected by the diaphragm and other muscles (inspiration). The subsequent elastic recoil (expiration) of the lungs and chest walls expels the impure air, warmed, moistened and charged with carbonic acid derived from the blood. Air as it enters the lungs consists of a mixture of nitrogen (N. 79 parts) and oxygen (O. 21 parts); as it leaves the lungs it is 4 per cent. poorer in oxygen and 4 per cent. richer in carbonic acid ($CO_2$). Respirations are to pulse as 1 to 4, *i.e.* about 10 to 15 a minute; but in disease they may be even 30 or 50 a minute (dyspnœa, orthopnœa). About 30 cubic inches of air are taken in at each respiration (tidal air); on an effort more can be expelled (reserve air, 100 c.i.); but some always remains in the lungs (residual, 100 c.i.). The amount of carbonic acid gas in the atmosphere is about ·04 per cent. If the amount added as the result of respiration or combustion be more than ·02 per cent., making a total of ·06 per cent., the air is becoming impure; the object of ventilation is to keep down the $CO_2$ in the air to less than ·06 per cent. To effect this, it is necessary for each adult to be supplied with 3,000 cubic feet of air per hour; and this can only be accomplished without undue draught by allotting about 1,000 cubic feet of space to each person. If insufficient oxygen be supplied to the blood asphyxia ensues upon dyspnœa, and unless speedily relieved will terminate in death. Artificial respiration, *i.e.*, imitating expansion and contraction of the thorax by external manipulations of the chest and arms, may relieve threatened asphyxia.

Hæmoglobin—the essential constituent of red corpuscles—is a red, crystallisable, albuminous substance possessed of the remarkable property of picking up and combining oxygen where this is plentiful, and disposing of it with equal readiness where oxygen is in demand; it changes its colour, and thereby the colour of the blood as the result of aëration from purple (venous) to crimson (arterial).

Respiration is sometimes spoken of as external and internal: external respiration is effected in the capillaries of the air cells which compose the two lungs; internal respiration takes place in the tissues of the body where by means of the systemic capillaries, the blood stream with its oxygen-bearing corpuscles is brought to the door, as it were, of the cells which collectively compose the body.

The right lung is larger and heavier (22 ozs.) than the left

(20 ozs.) and has three lobes, while the left has two. Healthy lung floats in water by virtue of its spongy, air-celled structure. Air enters the lungs through the mouth and nose, pharynx, larynx (organ of voice) and trachea. The latter is composed of some twenty cartilaginous rings incomplete posteriorly where the trachea abuts on the œsophagus or food-pipe. Some seven of these rings are above the top of the sternum; the isthmus of the thyroid gland overlies the second, third and fourth rings and immediately above (or below) this the operation of tracheotomy is performed. Laryngotomy is performed through the crico-thyroid membrane which connects the thyroid and cricoid cartilages of the larynx. The trachea is $4\frac{1}{2}$ inches long and bifurcates at the level of the third dorsal vertebra, *i.e.*, opposite the junction of the first and second pieces of the sternum into the right and left bronchi. The trachea and bronchial tubes are lined with mucous membrane covered with delicate ciliated cells (epithelium). Mucus may be secreted in excess as in bronchitis.

*Sputa* may be mucous, purulent, rusty (blood-stained), nummular, plastic. A lung or a portion of a lung may be rendered airless and consolidated by inflammation (pneumonia) or may undergo ulceration causing breaking down, bleeding (hæmoptysis) and formation of cavities as in phthisis. The component air-cells may be distended and dilated and fuse as in emphysema. The cavity of the pleura which in health is potential or whose surfaces are bathed with only sufficient serous fluid to effect lubrication may, as the result of disease, contain air (pneumo-thorax), blood (hæmothorax), or serum in excess (hydrothorax or pleurisy with effusion), or pus (empyema), or the surfaces may be inflamed and rough causing a friction sound to auscultation (dry pleurisy).

*(To be continued.)*

### The Training at Queen Charlotte's Lying-in Hospital.

A Chat with the Matron. By our Commissioner.

The exterior of Queen Charlotte's Lying-in Hospital has undergone little alteration in recent years, but many improvements have been made in the interior; and here as elsewhere the needs of the nurses have been recognised and provided for. When I had an interview with the matron last week she afforded me an opportunity of seeing precisely what has been done in this direction; and certainly it must be admitted that the institution in Marylebone Road, with its century and a half behind it, may safely challenge comparison with modern hospitals. The accommodation for the sisters and staff nurses is exceptionally good, as well as airy, the combined sitting and bed-rooms being of admirable size and furnished with much taste. There are also a general sitting-room, dining-room, box-room, and bath-rooms; while one commodious apartment on the floor at the top of the building is used by the pupils as a cloak-room. The pupils, of course, are housed in the handsome building opened two years ago by the Duchess of York, which I subsequently inspected, after I had been into some of the carefully kept wards, and the nice little chapel in which the chaplain baptises the babies.

### The Pupils' Home.

At the home, which has the advantage of being only across the street, the sister in charge met us and showed me over a portion of it—sufficient to justify the assertion that the inmates have no cause for complaint. There are no fewer than 70 bedrooms, each of which contains a fire-place, and every requisite convenience, and several sitting-rooms. The matron referred to the fact that before the home was erected the pupils had to sleep in the wards of the hospital. There was no other arrangement practicable in the circumstances, but it would be impossible to exaggerate the benefits of the improved order of things, both to patients and to pupils.

### Pupils increasing.

One result has been the increased popularity of the training school. During the seven years Miss McCord—who herself was trained at Crumpsall Infirmary, and has had a varied experience—has been matron, she has seen a considerable development.

"How many pupils did you find here when you came?" I inquired.

"There were under thirty. Now we have an average of about fifty, of whom forty are pupil nurses, and the remainder pupil midwives."

"When did the committee commence to take pupils?"

"More than twenty years ago."

"At what age are pupils received?"

"They must not be under twenty-three nor over forty. The average age is about thirty."

### The Training.

"And the period of training?"

"Three months, both for monthly nurses and for midwives The fees for the former are 15 guineas, and for the latter £26 5s. I am glad to say that the number who go in for the double course is increasing. In any case a pupil midwife is trained for three months in monthly nursing before commencing her course of instruction as a midwife. But when the double course is taken, the first three months are devoted to monthly nursing, and the second to midwifery. It is to the advantage of the hospital and of the public that both should be taken."

"You do not, I gather, think that three months' training is absolutely sufficient?"

"No; my view is that every monthly nurse ought to be a midwife, and every midwife a monthly nurse. I think the training of monthly nurses more important, and I should like to see every pupil trained for four months in monthly nursing and two months in midwifery."

"Just about the third month," continued the matron, "the pupils begin to be really useful, and then they usually go. But it would be for their good, as well as for ours, to remain."

"From what classes are the pupils drawn?"

"All classes. A fair number have been children's nurses, and when they are of a superior kind they make excellent monthly nurses. I suppose that about half the applicants for admission now are of the better class, and the proportion is still increasing."

### Lectures and Examinations.

"I should like to know what lectures are given."

"Two every week by the visiting physician, two by the house surgeon, and two by myself; also four clinical lectures."

"That is ten weekly."

"Yes; the pupils learn a great deal from the clinical lectures."

"Of course they are examined before they get their certificate?"

"Always, and unless the visiting physician who examines in writing and *viva voce*, is perfectly satisfied, the certificate is withheld. If, however, a pupil cannot pass the examination to obtain the certificate in midwifery, she is allowed to present herself for re-examination on a subsequent occasion."

UNIFORM AND FEES.

"Has uniform to be worn out of doors?"

"No; I prefer that it should not be worn out of doors, and it is also an unnecessary expense for short training. Indoors it is obligatory. The dresses of the indoor uniform are of white 'piqué," made quite plain, with tight-fitting bodices, with sleeves to unbutton to the elbow; the aprons of fine white linen, are very large, with square bibs, and straps across the shoulders.

"Is there any other payment except the fees and uniform?"

"Yes, each pupil has to pay the laundress, and she is required to provide herself with four dresses and twelve aprons."

HOURS OF DUTY.

"What are the hours of the pupil nurses?"

"For the first two months they are on day duty, commencing at seven and terminating at nine. They have two hours off daily, and a day once a fortnight. The third month they are on night duty from nine to eight, and that is uninterrupted, save for meal hours."

"How is the period of the midwifery pupils divided?"

"For the first month they are on day duty in the lying-in wards, and the second in the labour wards. The third month they work under the superintendent of the out-patient department and the resident qualified midwife of the district. Of course they do not go alone to patients; a midwife always accompanies them. The district training is an important feature; it gives the pupils a certain amount of confidence which otherwise it would be difficult for them to acquire."

"I conclude that you see all the applicants before they are admitted?"

"Yes; after they have sent in the form of application I make an appointment. Formerly they were accepted without an interview. As a rule, I find that they are suitable, though occasionally an application is refused."

HOSPITAL NURSES AS PUPILS.

"The difference in the training here and at general hospitals is very striking."

"Yes, we are continually changing. Sometimes people say that the fees are heavy. But bearing in mind the fact that a pupil is able to earn her living at the end of three months, if she is bright and industrious, I think that they are very moderate. We train upwards of two hundred every year."

"Are many of them hospital nurses?"

"A considerable proportion. I wish that they were all trained nurses. But that cannot be, and sometimes we get a stupid trained nurse, just as we often get a bright pupil who has had no hospital training. All the pupils live here and sleep at the Home."

THE STAFF.

"How large is the regular staff?"

"There are seven sisters and five staff nurses."

"All fully trained?"

"Yes, all have had three years' hospital training, and most of them have been sisters or staff nurses before they came here. As to their duties, two of the sister midwives superintend the labour wards, one being on duty in the day and the other at night. There are four sisters in charge of the lying-in wards—one acting as night superintendent, while the other three are in charge of the three floors by day. They have four hours off duty every day, one day a month, three weeks' holiday, and frequently an extra week."

"And the staff nurses?"

"One is on duty with each sister during the day, and one with the night sister. The staff nurses get the same holidays as the sisters, and the same time off duty, except that they have four hours off one day and two the next. Neither sisters nor staff nurses need wear outdoor uniform."

"How about the midwifery training of the sisters and staff nurses?"

"I often have to train them in midwifery after they join the hospital, and in some cases have done so with great advantage."

THE REGISTRATION OF MIDWIVES.

"I conclude that you will be glad when the Midwives' Bill becomes law?"

"Very, as I am sure that it is always better for people to have the services of a trained than of an untrained midwife. Moreover, registration will, in my opinion, improve the training generally, and tend to increase the number of educated midwives."

"You know that sometimes midwives take upon themselves to give certificates of death. There was an instance the other day."

"I can only say that such a thing is very wrong as well as entirely contrary to law. We are most particular in pointing out that our certificate does not entitle a midwife to undertake the medical treatment of cases, nor the management of complications in labour."

### Appointments.

BEXLEY COTTAGE HOSPITAL.—Miss Susie Dunham has been appointed nurse matron. She was trained at the London Hospital, and has since been staff nurse in the same institution.

LANCASTER SANATORIUM.—Miss Madeleine Aspinwall has been appointed charge nurse. She was trained at St. Mary's Hospital, Paddington, and was afterwards charge nurse at the South-Eastern Fever Hospital, London, and sister at the City Hospital, Sheffield.

MAIDSTONE FEVER HOSPITAL.—Miss Annie Brandon has been appointed assistant matron. She was trained at Monsall Hospital, Manchester, and has since been assistant matron at the Sanatorium, Mill Hill, Dalton, Huddersfield.

MACCLESFIELD GENERAL INFIRMARY. — Miss Eleine Traiforos has been appointed matron. She was trained at the Victoria Hospital, Burnley, and has since been sister at the Royal Infirmary, Aberdeen, and matron at the Memorial Hospital, Mirfield.

MANSFIELD AND MANSFIELD - WOODHOUSE DISTRICT MEDICAL HOSPITAL AND CONVALESCENT HOME.—Miss Mary Bayldon has been appointed matron. She was trained at the Royal Infirmary, Hull, where she was afterwards staff nurse, charge nurse, private nurse, and night superintendent.

SKIPTON AND DISTRICT HOSPITAL.—Miss Jessie B. Taylor has been appointed staff nurse. She was trained at Leeds Infirmary.

ST. GILES' INFIRMARY, CAMBERWELL.—Miss Bessie Baker has been appointed sister of the women's medical ward. She was trained at Guy's Hospital, where she was head nurse for a year in Bright ward.

ST. OLAVE'S UNION, LADYWELL, S.E.—Miss Street has been appointed superintendent nurse. She was trained at St. George's Infirmary, Fulham Road, where she remained as staff nurse eight years. She has since been charge nurse at St. Olave's Infirmary, Rotherhithe, for nine years.

### Presentations.

CITY HOSPITAL, BRADFORD.—Last Wednesday a social gathering was held by the nurses of the City Hospital, Bradford, in order to present Miss Hewer with a handsome silver-mounted dressing-case, on the occasion of her leaving to be married to Dr. Manknell. The chairman of the hospital committee referred to the long service Nurse Hewer had rendered on the staff, and the presentation was made by Miss Mellor, the matron. A most enjoyable evening was afterwards spent. On Friday the matron entertained the nurses to supper in honour of the same occasion.

### How Smallpox was Nursed at the Belvidere Hospital, Glasgow.

By One of the Staff.

Now that the epidemic of smallpox in Glasgow is happily so far over, a few words regarding the nursing of the patients in Belvidere Hospital may be of interest to the readers of "The Nursing Mirror."

The incubation period of smallpox is from ten to thirteen days, usually about twelve. The first symptoms are shivering, headache, sickness, and severe pain in the back, accompanied with high temperature. On the third day the eruption comes out. Before its appearance it may be felt like "shot" under the skin, and there is frequently an erythema which may be mistaken for scarlet fever or measles. After the eruption is out the patient feels well, and in a mild case this is usually the end of his troubles.

### Severe Cases.

In a severe case it is quite the reverse, as about the seventh day of illness the secondary fever begins and the patient again becomes very ill. The eruption may become confluent and the patient be almost unrecognisable. Owing to the swelling of head and face the eyes are closed up and loss of sight is to be dreaded. Pustules are seen on the soft palate which give rise to sore throat. The smell of a bad case is most offensive and loathsome. Delirium is of an active character, and the patient suffers from delusions. The complications are pneumonia, laryngitis, and inflammation of the eyes. Boils are also frequent. Albumen is present in the urine, but disappears as the patient improves. In malignant cases the eruption is undeveloped and of a hæmorrhagic character. There is bleeding from the mucous membrane.

Most nurses, though hardened to the sight of disease in all forms, will experience a sort of shock on entering for the first time a ward filled with smallpox patients. Especially is this so when the patients are in the acute stages of this dreaded disease. The loathsome smell which is peculiar to smallpox also adds to the unpleasantness. But whatever feelings of disgust and horror a nurse may for the moment undergo, they are quickly lost in the sense of compassion and the longing to do something to relieve. And there is much that can be done by a nurse to alleviate their sufferings.

### Mild Cases.

During the late epidemic, the mildest to the most malignant forms of the disease were admitted to the Belvidere Hospital. Smallpox is frequently spread by mild cases, as the constitutional disturbance may be slight, and the eruption escape notice altogether. These mild cases were undoubtedly a fertile source of infection during the last few months. When a mild or modified case was admitted to the hospital little treatment was required beyond the utmost cleanliness and the placing of the patient in the most favourable circumstances tending to recovery. He was promptly bathed and put to bed. His hair was cropped or shaved. He was allowed to have light, nutritious diet—milk, chicken soup, chicken, fish, &c. If his eyes were affected they were bathed with boracic lotion or McKenzie's eye lotion. Crusts were softened with carbolic oil 1-40. When they began to come off his body the doctor gave orders for him to be bathed once daily for three days. At the end of this time if his body was found to be free he was allowed to dress and sit up. Until his dismissal, which took place about the end of twenty-eight days, if all went well, he took a bath daily, using a flesh brush. This bathing was a source of wonder to many of the patients; several being heard to say they had never been so clean before. One old man was amazed at the number of clean shirts he got, and could not imagine why the bed linen was changed so often. "My auld wife," he said, "only pits on clean sheets when she's expeckin' the minister to gie's a ca'." Who says respect for the cloth is dying out in old Scotland?

### The Value of Vaccination.

This modified form of the disease was usually found in those who had been successfully vaccinated, for though vaccination does not render one immune it may be said to ensure a very mild attack, and it also reduces the chances of a person contracting it. Many were the regrets of patients that they had neglected to be revaccinated. One man was very anxious to know if his brother had got revaccinated. "If he hasn't," he remarked, "a sight of the poor objects here would soon convince him that vaccination is the only remedy." Indeed, this opinion was shared by all.

### The Treatment of Severe Cases.

In severe cases bathing on admission was impossible, and washing down in bed had to be done very gently owing to the profuse eruption and consequent tenderness of the skin; sometimes it had to be dispensed with. The hair, if possible, was shaved, but where this was impracticable, owing to the eruption being too far out, it was closely cropped. The patient wore a cotton gown and lay between sheets. On no account was he allowed to sit up. The forehead and face were frequently painted with carbolic oil to remove the feeling of tension. The eyes were kept covered and were bathed every two hours or so with boracic lotion or McKenzie's eye lotion. Compresses were often applied. The mouth, which was exceedingly dirty, with the tongue very dry, was washed out with chlorine water and glycerine of borax used. The throat was swabbed with carbolised glycerine. Frequently the nostrils had to be douched. For sore throat and laryngitis Maw's inhaler with boiling water simply was found to give great relief. The back and front of chest were dusted with boracic powder. In cases where the smell was very offensive, careful sponging with a weak solution of carbolic lotion or Condy's fluid added much to the patient's comfort as well as to that of others.

### The Extreme Discomfort.

Unless one has seen a bad case of smallpox it is hard to realise the extreme discomfort and uneasiness from which a patient suffers. The throbbing and swelling of the limbs and the tenderness of the whole surface of the body are almost unbearable. The limbs in some cases had to be dressed several times a day, especially where there was much suppuration and consequent discharge. The dressings varied: sometimes vaseline and eucalyptus was ordered or carbolic oil. Where a raw surface was left Hay's wash was sometimes employed or a weak solution of carbolic. Boracic acid ointment was also used. The patient's feet were relieved of the weight of the bedclothes by means of a cage, and left uncovered, which helped to keep them cool. A pillow was put under them, elevating them, and thus relieving the throbbing. Even with the utmost care, bed sores were met with, as can be imagined, owing to the state of the skin. Abscesses were treated with antiseptic dressing, and the other complications as they arose.

### How Strength was Maintained.

The strength of the patient was kept up with milk, egg flip, chicken tea, meat juice, &c., and brandy and whisky were the stimulants given. Lemonade was given as a refreshing drink.

In cases where there was much sickness iced champagne and peptonised milk were well retained. Opium in the form of Battley's solution was the usual drug administered for sleeplessness. When convalescence started port wine was sometimes ordered; later on stout was given in cases of emaciation.

The wards were kept at a temperature of about 60°, and thorough ventilation was enforced.

Smallpox Fingers.

Every nurse was revaccinated, even though she had been vaccinated within the last few years. On entering the ward she donned a holland overall which completely covered her uniform. This was removed on leaving the ward. Personal hygiene was carefully attended to. Several of the nurses had suppurating fingers which sloughed and were troublesome to heal. These got the name of "smallpox fingers." Otherwise the health of the nursing staff was remarkably good.

### Asylum Workers: Annual Meeting of the Association.

### SIR JAMES CRICHTON-BROWNE ON NURSING THE INSANE.

The meeting room at the Medical Society's House, 11 Chandos Street, W., was well filled on Friday afternoon with members and friends of the Association of Asylum Workers, on the occasion of the annual gathering. Sir James Crichton-Browne, President of the Association, was in the chair.

In moving the adoption of the fourth annual report, the Chairman said that his remarks would be chiefly addressed to the rank and file of asylum workers. He called attention to the quadrupling of the number of members of the Association in one of the county asylums, owing to the energy of the local secretary, and said that if the organisation was to be really perfect, every lunatic asylum in the country must have a local secretary. Perhaps some badge of office might be conferred. It was most satisfactory to note the increasing interest taken in the Association by asylum workers in the Colonies, which made the prospect of an international alliance seem not impossible. Dr. Greenlees, medical superintendent of Grahamstown Asylum, was now in this country, and he hoped that through his help many valuable openings might be found in South Africa. He trusted that when this grievous war was over, and those members now serving in South Africa returned, they would have a very cordial reception; their places were being kept open, and it was right that the time thus spent should count for their pension.

The Financial Position.

Financially, the Association was in a satisfactory condition. At the end of 1890 the credit balance was £153 17s. 10d.; at this rate the Association would be very rich in the course of a century or two; nevertheless, if Mr. Carnegie had another million to spare, it would not be refused. With regard to legislation, he did not think a Bill would pass at this stage of the session; but, thanks mainly to the energy and foresight of the hon. secretary, Dr. Shuttleworth, the Association was in a much stronger position for making its wishes known, and a large number of members of Parliament were pledged to support a pension clause when the Lunacy Bill should come before the House.

The Strain of the Work.

The Home of Rest Fund was very greatly appreciated; the conditions of asylum life were such that cases of nervous breakdown were far more frequent than most people imagined; and these had an especial claim on the fund. How many, he wondered, broke down altogether, and finally; and how many never let it be known that they had broken down? How many carried on their work under a load of misery? The strain was a terribly severe one, and was not by any means appreciated by the public, who based their opinions on a patients' dance, or a stroll through the wards on a sunny afternoon. Every advance in the scientific treatment of insanity tended to aggravate the nervous strain on the nurses and attendants, and this had not yet been alleviated by shorter hours or increased facilities for recreation. The workers were, moreover, of a much more nervous class than they used to be. When wages were lower, and a prejudice existed against asylum life, they were drawn from a lower social scale, in which there was more solidity and greater power of resistance. Now, what was required in an asylum nurse or attendant was intelligence and vivacity, the faculty of sympathy with brain-disorder—in fact, a highly nervous organism; and this organism was made more nervous by training. It must not be supposed that he found fault with these changed conditions—he highly approved of them; but the system had its drawbacks in the increased neurotic condition of those employed in the care of the insane. The standard of work was much higher, and involved a greater strain.

Then and Now.

He had heard of a preacher who asked his congregation whether they thought Adam and Eve went about with their hands in their pockets. Thirty or forty years ago asylum workers did occasionally do so, but now the round of duty required incessant vigilance; some clinical observation was necessary, and with the power of sympathy and pity, unremitting self-control. The consequent nervous exhaustion was not surprising. There were, too, other and more conspicuous hazards—risks of infectious diseases, jeopardy of life and limb—and courage equal to any displayed in the siege of Mafeking was frequently shown; courage which was not stimulated by the prospect of the Victoria Cross, a banquet, or even a modest annuity. Among those who had benefited by the Home of Rest Fund was a nurse who had sustained in the course of her duties at a county asylum an injury to the knee, which necessitated amputation of the leg; and one of H.M. Commissioners in Lunacy had received a stab from a patient while performing his duties. As Lord Chancellor's Visitor in Lunacy he (the Chairman) could look back on a long list of disablements; the asylum worker's life was not a path of roses.

The Compensations.

But there was a compensatory side; there was steady employment, and, in short, punctual payment, usually a considerate employer who safeguarded their interests, and, above all, they had a mission. It would be difficult to exaggerate the influence of a good nurse or attendant; many a brain gone wrong had been righted by the gentle rain of sympathy; a nurse might wield a power almost as healing as that of drugs. He had constantly seen cases regarded as incurable—even "chronic" cases—in which recovery had taken place; he had seen the film fall from the mental vision, and the darkened chambers of the brain lightened. Nurses should maintain a cheerful attitude, and regard none as debarred from the hope of recovery.

Dr. Jones (Claybury) seconded, and the report and accounts were unanimously adopted.

Mrs. Chapman, formerly matron of Leavesden and Claybury Asylums, was appointed hon. treasurer to succeed Miss Evans, Miss Honnor Morten having temporarily undertaken the office, but being unwilling to be appointed permanent treasurer.

### To Nurses.

**We invite contributions from any of our readers, and shall be glad to pay for "Notes on News from the Nursing World," or for articles describing nursing experiences, or dealing with any nursing question from an original point of view. The minimum payment for contributions is 5s., but we welcome interesting contributions of a column, or a page, in length. It may be added that notices of entertainments, presentations, and deaths are not paid for, but, of course, we are always glad to receive them. All rejected manuscripts are returned in due course, and all payments for manuscripts used are made as early as possible after the beginning of each quarter.**

### West Ham Typhoid Epidemic.

By a Correspondent.

NURSING IN THE HOMES.

THE local hospital accommodation being quite inadequate to meet the demands of the epidemic of enteric fever which has broken out so suddenly in the adjoining districts of West Ham and Canning Town, the borough authorities were obliged to take other steps to secure efficient nursing of the cases and to prevent the further spread of the disease.

A Chat with Dr. Sanders.

In order to ascertain exactly what these arrangements were, I visited the medical officer of health for West Ham, Dr. Sanders, who readily gave me all the information in his power. Dr. Sanders has circulated a simple but cogent list of precautions, which is to be found in all the houses where there are cases of typhoid, or suspected typhoid, but the intelligent carrying out of these directions is chiefly effected by the active supervision of the nurses working in the homes.

Where did these nurses come from was naturally the first question to be asked. "Some," said Dr. Sanders, "have been sent down from London, but the majority are attached to the Plaistow District Nurses' Home, under the super-intendence of Sister Katharine, in whose hands I have entirely left the management of the nursing."

"How does Sister Katharine hear of the cases?"

"I send her a list of the fresh ones every morning, and she puts her nurses in at once, visiting the cases herself."

"But do the patients have a nurse with them always, or are they visited as district cases?"

"That is arranged according to their condition. If they require night nursing, they have it. Sister Katharine has *carte blanche* from me to do whatever is necessary, and from my own observations and the reports of my inspectors every-thing is being done excellently."

Having thanked Dr. Sanders for his courtesy, I proceeded to act on his suggestion of a visit to Sister Katharine, and found my way to Howard's Road, the central home of the Plaistow District Nurses. Here was a scene which reminded one of a beehive just before swarming time. It was just the hour for the departure of the nurses on their evening round. Nurses, bicycles, bags and buckets seemed to be everywhere in the continuous passing to and fro beneath the porch, and every now and then a maid would stagger out with a bundle of bedding, crockery, and the like for loan amongst the patients.

Sister Katharine's Campaign.

Despite the pressure of business, rendered more acute by the building operations still going on in the new extension, Sister Katharine (Miss Twining) not only made me welcome, but suggested that, for the purpose of my inquiries, I should witness some of the actual work in the district, escorted at first by a nurse on the point of starting on her round, and later on by herself. It was during this second series of interesting visits that Sister Katharine gave me the details of her organised campaign. Eighteen of her own staff nurses had been told off for typhoid work with a certain number of assistant nurses from the home to work under them, each staff nurse being made responsible for six cases. Besides these, in reply to Sister Katharine's applications, six nurses were sent down from Guy's Hospital, a few from St. Thomas's, St. John's House, and one or two other institutions, and four were expected down that night from the "London." These hospital nurses are chiefly doing night duty, the cases being visited during the day from the Plaistow Home, the regular inmates of which naturally know their way about the district better than the visitors. The Home, it may be imagined, is packed full by reason of the temporary increase in its numbers, and the domestic staff has been added to and reorganised, everything not essential being put aside to ensure the comfort and welfare of those engaged in the arduous fight with the epidemic. The nurses who are told off for typhoid work call it "going to the front," and shirk no part of their often unpleasant duties, which, one might almost say, include scavenging and house-cleaning. In the opinion of the Lady Superintendent this method of dealing with the contagion is likely to prove a most valuable object lesson to the inhabitants of the district, and an effectual means of securing greater domestic cleanliness hereafter.

How the Patients are Treated.

Armed with a list of the rules drawn up for the nurse's own guidance and a separate slip for the instruction of the patients' friends (a copy of which I found pinned to the wall in each house), I started forth with my guide, and the best idea of the methods adopted may be gathered from a brief description of what I saw. The buckets previously mentioned, which resemble wooden milking-pails, are used to contain the disinfectant which is kept in the room of each patient, and stringent rules oblige the nurse to place therein all personal and bed linen immediately on being removed from the patient before it is taken from the room. After the usual preliminaries of taking pulse, temperature, and respiration, which are charted for the doctor's information, a patient is washed and changed and the bed is made, the slops then being emptied by the nurse herself, who takes this opportunity of flushing the drain with disinfectant, inspecting the backyard, the dustbin, and the cistern, directing, and if necessary assisting in the all-important matter of getting these places into a sanitary condition. This inspection is carried out twice a day. Every part of the inside of the house is also visited and the patient's relatives are persuaded to scrub with disinfectant and thoroughly clean walls, floors, staircases, and cupboards. In the sick room itself all carpets and unnecessary draperies are removed and absolute cleanliness is insisted on. Papers for the doctor's orders are pinned to the wall close to the chart and according to the directions written thereon tepid sponging is performed, food and stimulants given, &c. Of course it has been necessary in many cases of extreme poverty to supply all the bed linen, as well as nursing appliances, such as macintoshes, feeders, and bed-pans. Even bedding has in many cases been included in the loans, impromptu mattresses having been manufactured by filling unbleached calico with clean wood shavings (a gift of the Thames Ironworks Company) which are afterwards easily destroyed.

The Worst Apparently Over.

Sister Katharine herself visits the worst cases every day, taking the fresh ones sent in by the medical officer in the morning, and those already on the list in the evening, when she sees and superintends the work of the nurses, making herself acquainted with the individual requirements of the patients. In her company I saw three children in one room, two of whom had had a severe form of the disease, but had now turned the corner. Here the room had been entirely rearranged, and a cot lent, and sister was asking for books and magazines to keep the juvenile patients quiet during the tedious time of convalescence. In many other houses there were two in one room, but in spite of the very limited space and other notable disadvantages, it was encouraging to observe that most of the cases were on the way to recovery, even after periods of great danger. The fact that the number of fresh cases is already reduced to five a day—a fact indicative of the arrest of the spread of contagion—speaks eloquently for the success of this highly-organised effort to combat a serious public danger.

### Everybody's Opinion.

[Correspondence on all subjects is invited, but we cannot in any way be responsible for the opinions expressed by our correspondents. No communication can be entertained if the name and address of the correspondent is not given, as a guarantee of good faith but not necessarily for publication, or unless one side of the paper only is written on.]

### PREACHING AND PRACTICE.

"TIRED NIGHT NURSE", writes: Here is a question on which I should like the opinion of your readers. I am night nurse in a small hospital, hours on duty 10 P.M. till 9 A.M. The hour for the sister (to whom the report must be given each morning) to come on duty is 9 A.M. Sister never comes on duty before 10 A.M., often as late as 10.25 A.M., thus causing the night nurses to lose at least one hour off-duty time. Punctuality is enforced on the nurses by the sister, yet is it right that she should demand obedience in this particular while she herself habitually breaks the first rule of the day?

### A WARNING TO MATRONS.

"M. P. C." writes: Seeing "A. B.'s" letter, I should like to state that a person answering to the same description called at a suburban nursing institution in the south-east of London on the morning of April 5th last. She said that her brother had been wounded in South Africa, and was expected home next day, and that it was necessary for him to undergo an operation. She therefore made all necessary arrangements for his admittance. As she was about to leave, she explained that another brother had taken a single ticket for her, and as she had forgotten her purse she had no money to return to town. On these grounds she received a small sum, which she promised to return the same afternoon. However, nothing has been heard of her nor of the supposed patient from that time.

### SELECTION OF NURSES FOR PLAGUE DUTY IN SOUTH AFRICA.

"A PLAGUE NURSE" writes: The subject I am about to touch on is one of extreme delicacy, but nevertheless it is of the utmost importance that it should be brought to the notice of our matrons, &c., if we are to uphold the honour and purity of our profession. I mean the personal character, apart altogether from the professional ability, of the nurses whom matrons recommend as suitable for duty abroad. I myself form one of a total of twenty nurses who have been sent out to South Africa to nurse the plague, and from the day of embarkation at Southampton till the day of arrival at Capetown the majority of us have had only too good reason to wish that women of a higher type had, in one or two instances, been chosen to represent our profession. I know of no surer test of a woman's character than her behaviour on board ship, and to those of us who are jealous of the honour of the nursing profession nothing can be more humiliating than to see nurses behaving in a manner, not only totally unworthy of those who have given up their lives to the nursing of the sick, but also in a way calculated to bring discredit on womanhood itself. Surely it is the duty of our matrons to think well before they recommend women for duty in foreign countries—women in whose hands is placed the honour of our beloved profession—if these hands are not perfectly capable of upholding and shielding it. The world at large is quite ready to yield all due respect to nurses; but if nurses themselves are so utterly lost to all sense of what is due to their profession—not to say what is due to their sense of womanly dignity—as to allow men who are total strangers to them to put their arms about them, &c., then surely it is the wrong kind of nurse who has been sent. There is no reason whatever why nurses should not thoroughly enjoy the voyage to the scene of their work (in most cases they have more than earned a holiday), but this I do think, that it is only nurses who are entirely to be depended upon as regards discretion and good taste who ought to be sent abroad. I do not know if more nurses will be sent out to the plague on this occasion, but, if there should be, I am sure I shall have the whole nursing world with me in hoping that only women of the highest moral tone may be selected.

### Examination Questions for Nurses.

### PREVENTION OF SPLINT SORES.

RESULT OF MAY COMPETITION.

THE question was as follows:—"In a case of fractured thigh treated with a long splint, in what places should you watch for splint sores, and what means should you take to prevent them?"

NO PRIZES AWARDED.

It is a sad fact that not a single paper sent in this month deserves a prize. The cause for this falling off in good work is a mystery, for the question is not so difficult as many that have been answered most successfully.

HONOURABLE MENTION.

The two best papers out of a large number of very indifferent ones belong respectively to "Nurse Percy Vere" and "Nurse Manningham" and they will be awarded honourable mention.

CHIEF DEFECTS IN THE PAPERS.

Several candidates state with finality that with a well-padded splint there should be no danger of splint sores; one might as well say that with a well-washed back there should be no danger of bed-sores. Hospital experience shows that however well padded a splint is, there is always danger of pressure sores, a danger that should be always present to the mind of a capable nurse. Some conditions of skin are such that the mildest pressure, even such as that caused by a flannel bandage (put on lightly) over the knee-cap is sufficient, if not closely watched, to produce a sore. A large proportion of the candidates make the common mistake of suggesting wool pads where the pressure is felt. This adds to the mischief already set up by making the pressure greater; the only way successfully to obviate pressure is to put a pad above the desired spot and below it, so as to leave the tender surface free and not heated.

The danger (very important) of sores resulting from strapping used in fastening an extension is noticed by several, I am glad to see; it is a serious difficulty and one to which constant attention must be given. This question will be given again in the autumn and I hope with much better results.

THE QUESTION FOR JULY.

How should you arrange treatment to a leg by constant irrigation if in an outlying district where the proper appliances were not to be had?

N.B.—This will be the last examination until October.

THE EXAMINER.

RULES.

The competition is open to all. Answers must not exceed 500 words, and be written on one side of the paper only. The pseudonym, as well as the proper name and address, must be written on the *same* paper, and *not on a separate sheet.* Papers may be sent in for fifteen days only from the day of the publication of the question. All illustrations strictly prohibited. Failure to comply with these rules will disqualify the candidate for competition. Prizes will be awarded for the two best answers. Papers to be sent to "The Editor," with "Examination" written on the left-hand corner of the envelope.

N.B.—The decision of the examiners is final, and no correspondence on the subject can be entertained.

In addition to two prizes honourable mention cards will be awarded to those who have sent in exceptionally good papers.

### Death in Our Ranks.

MISS AGNES G. STEVEN died on April 9th, after a few days' illness, at Pernambuco, where she was nurse-matron at the small English hospital. Having received her training at the Royal Infirmary, Glasgow, she was afterwards charge-nurse at the South-Eastern Fever Hospital, London, and sister at Monsell Fever Hospital, Manchester. She was also for some time on the staff of the Nursing Institution at 31 Catharine Street, Liverpool.

### Wants and Workers.

WOULD any nurse like "THE HOSPITAL" not later than the Wednesday after publication? If so, she can have it by sending stamped and directed wrapper to III., 23 Cleveland Square, Hyde Park, W. Please send postcard first.

### Echoes from the Outside World.

AN OPEN LETTER TO A HOSPITAL NURSE.

WE went to the opening of the Naval and Military Exhibition at the Crystal Palace. Long before the time had arrived for Lady Roberts to unveil the statue of her husband every approach to the central nave was densely filled, and some of the spectators realised more than they desired that summer had come, as the sun poured down steadily through the glass. When at last the period of waiting was over, and Earl Roberts, with his wife, came in sight, for a moment a feeling of disappointment took possession of some of the ladies, that he had not appeared in the glory of full regimentals, and I heard some one murmur disconsolately "Why, he's only got on a black frock coat just the same as everyone else!" Lady Roberts—she has such a kind, motherly face!—wore black silk and was presented with a patriotic bouquet of red, white and blue, the same colours being again represented in the cords and tassels with which she pulled aside the draperies concealing the statue. As she did so the band struck up "See the Conquering Hero Comes," and the green bronze figure of "Bobs," upon his charger, was revealed. A replica of the statue, I learn, has been sent to India. At the formal opening ceremony Lord Roberts alluded to the day when he visited the Crystal Palace, and saw, for the first and last time, the great Duke of Wellington. It was a particularly happy allusion, linking the past with the present, and suggesting a vivid picture of the young lieutenant, eager for experience, just on the eve of his departure for India, receiving, as it were, a fresh access of enthusiasm and courage from his glimpse of the veteran soldier.

AFTER the ceremony was over came the inspection of the boys from the training schools, and dapper little fellows they were in their clean white and blue suits, with a look on their faces of earnest endeavour mingled with proud satisfaction that the Commander-in-Chief should come to see them drill. Then followed the final unrehearsed incident, when the horses attached to Lord Roberts' carriage took fright and charged a fountain, demolishing it, and doing damage not only to it and themselves but also to others in the crowd. A spectator did much to stop the horses' mad career by springing at the reins, and fortunately no serious accident occurred; but at one moment it seemed as if both Lord and Lady Roberts might be thrown down by the violent surging of the crowd as it strove to get away from the plunging horses. Lord Roberts wisely declined to enter the second carriage brought up for him till it had been driven outside the grounds. As soon as the guests of the afternoon had departed we had a peep at the Exhibition itself, but there was so much to see that it was impossible to take notice of half the exhibits, and a second visit will be needed later on. The most interesting collection has been got together, and the show, more especially just now, is bound to be as popular as it deserves. I must mention that the care taken of the wounded in war is admirably illustrated. There is a field hospital, with collecting and dressing stations and various methods of transport, including an ambulance train, and a full-sized representation of a section of the "'tween-decks in the Princess of Wales's hospital ship." Also a model ward of Netley Hospital. The King's loan consists of the two Jubilee gifts given to Queen Victoria by the Navy and Marines, silver models of the *Victoria* and *Britannia* floating on a sea of the purest crystal. The German Emperor has promised to send a model of his yacht *Hohenzollern*. There are, too, the cloak Wellington wore at Waterloo, the trumpet which sounded the charge of the Light Brigade at Balaclava, and the spear which killed General Gordon in the Soudan. But perhaps one of the most interesting items will prove to be the military balloon used in Ladysmith to watch the besieging Boers until there was no longer gas to fill it. There are about forty neat little patches on it where it had to be repaired after being injured by Boer ammunition.

THE man of the hour is unquestionably Sir Alfred Milner. or, as he will henceforth be called, Lord Milner of Capetown. His peerage will be memorable in history as the first conferred by King Edward VII. The High Commissioner of South Africa is personally unknown to the masses, and when Lord Milner drove up to Claridge's Hotel on Saturday in a cab—the last of the guests to arrive—he passed in unnoticed. He seemed considerably overcome by the heartiness of his reception in the room, and after lunch said very nicely that to tell the honest truth he was rather ashamed to be where he was when there was "a big unfinished job" awaiting him. Later on, whilst he asserted that all the praise and the honours bestowed upon him were beyond his deserts, he wisely added, "But the simplest thing to do under these circumstances is to try to deserve them in the future." And those who know Lord Milner best say that that is exactly what he will do.

FOR once the holiday-makers were able to rejoice in a most ideal Whit Monday, and many of us basked more or less in the glorious sunshine. But ever and anon I found my thoughts wandering towards that scene of sadness in South Wales where, alike through the hours of sunshine and darkness, in spite of foul air and masses of dangerous obstruction, the work of rescuing the dead from the Universal Colliery at Senghenydd went on. Almost the only cause for thankfulness—apart from the fact that there are always brave men waiting to gladly undertake even such perilous tasks as the descent into a coal mine after an explosion—is that all the colliers seem to have perished at once and did not live on for days, as has sometimes been the case, starving slowly to death. Out of the 82 men who went down on Friday, only one has been rescued alive, and he, poor fellow, very nearly shared the fate of his comrades. He is a haulier, Harries by name, and he was found by the rescuer lying with his head on the breast of the horse of which he had charge. He was terribly burnt, the skin peeling off his face and hands. All the members of the rescue party thought him dead, but suddenly as they were discussing the question, Harries, though unable to speak, raised his hand. As it was impossible to send him to the surface at once, two doctors descended the shaft to his assistance. Since his rescue he has been delirious nearly all the time, and it is thought that he will never be able to give the slightest explanation of what occurred in the colliery on the morning of the explosion. A message of sympathy and condolence from His Majesty was amongst the first received by the authorities, and even those who had been the most severely stricken naturally felt glad that the King had not forgotten them in their sorrow. The mine is a new one, having only been opened a few years back. A very large proportion of the dead were married men, and one leaves a widow and eleven young children. Two poor little children lost both father and mother the same day. The mother died at the infirmary only a few minutes before her husband too was called away. Truly the price we pay for coal is sometimes heavy indeed!

ARMY nurses will learn with interest that there is to be a new Director-General of the Army Medical Department, in consequence of the retirement this week of Dr. Jameson, which is due to the operation of the age limit. There are rumours of a sensational appointment, but as there are plenty of suitable men available among the members of the Army Medical Corps it is highly improbable that an outsider will be chosen. Dr. Jameson, who has seen 44 years' service and has been indefatigable in the discharge of his duties, has always been most courteous to all who have come in contact with him.

### A Book and its Story.

### A DISCIPLE OF MEREDITH.*

By admirers of George Meredith *The Column*, by Charles Marriott, will be read with unabated zest. To readers unable or unwilling to appreciate the particular characteristics of the Meredithian style, the book will have neither significance nor charm. Mr. Marriott is so frankly a follower of his master that one cannot seriously cavil at a point which in less able hands might be a glaring defect. In bringing as he does, so much individual talent to the treatment, one only wonders why he could not trust to his own initiative, without showing so clearly the source of his style. The book has humour, has pathos, and the characters arrest by their unorthodoxy, and they will hold the reader who finds in this latter trait a guarantee for the absence of commonplace whether in incident or individual delineation.

The Doric column from which the author takes his title had been brought from Greece and placed by the heroine's father, Edward Hastings, on a headland overlooking the sea bordering the grounds of his Cornish estate. Here, surrounded by a circle of laurels, the mature successors of the slender cuttings planted by Hastings upon his return from the country of his adoption silhoutted against the sky, stood the slender shaft rising from the grove, a landmark to passing vessels, and a source of inspiration to the heroine in moments of mental exaltation, or depression. Savant and enthusiast, Hastings had found in Greece a kindred atmosphere. From youth he had been a traveller, his parents having died when he was in infancy—his father from a violent death by his own hand, and his mother upon the birth of her child. Their marriage was a romantic one, with unusual events following immediately on it. His mother, from whom he inherited his small estate, was the Countess of Tregotha, and his father "a Yeoman who read Virgil." A childhood of isolation, spent under guardians, ended upon his majority in his declining to settle in the corner of Cornwall, in which Penresco, his place, was situated. "So for twenty-eight years it had been given over to the sea-mew." When he returned to take up his position as owner, he was a widower with one child, Daphne, who was nine years of age. Some natural curiosity was excited in the neighbourhood by his return. But the almost recluse habits which distinguished Hastings did little to gratify those who, knowing something of his antecedents were ready to weave rumours of eccentricity or aloofness. "But most people, as time passed and nothing occurred to justify these suggestions, were compelled to take him on his own merits which were socially inoffensive enough. He neither shunned companionship with his kind, nor courted it with alluring eccentricities. His peculiarities, such as they were, being in the direction of his daughter's education, which aimed more at physical development than is usual at the present day. Except on this one point, the people of Tregotha described him as commonplace. And as he suggested their own mental superiority he was mildly popular, his irony being turned inwards." One "understanding" and congenial neighbour was found in the caustic vicar of the parish. In their mutual reverence for the Classic past they were on congenial ground. "Priest and pagan, equally honest and in earnest, met and mingled their enthusiams upon the soil of Athens." Herbert Waring, Tregotha's parish priest, is a delightfully unconventional character. His advent into its midst had been succeeded by storms of no common order. This in no way disconcerted him, being a man of methods and determination. He had succeeded a predecessor of an opposite type, and here, he decided, was his opportunity. The place was inhabited by two classes—Methodists, who were outwardly smooth and sanctimonious, and church-goers, who were very much the reverse. He won his way in the first instance by sheer arrogance, not, happily, unsweetened by a sense of humour." When storms arose he went calmly on his way, in spite of the tempest raging in parochial circles—which he was the cause of. Upon the Bishop remonstrating with him he replied, "My lord, the church has been likened to a ship; moreover Tregotha is a fishing village. Will you not therefore grant me the discretion of a captain, even in the matter of rope's-ending?" "But," said the bishop, "I hear of unnecessary candles—of incense." "My lord," replied Waring, "give me a year and you will hear no more of candles, nor even of incense." . . . "In twelve months Waring had Tregotha under his thumb. Where he was not worshipped he was dreaded. He took the men by sheer fellowship, and the women—by holding his tongue." The parish had its doctor, of course, and "Caspar Gillies, the professional cynic whom chance had made a village doctor" and the conductor of its amateur band, affords some excellent fun. The chapter "A Little Music" is really humorous. "The rehearsals took place in the village school-room; and Herbert Waring became almost human on the 'cello. With his Napoleonic eye for the uses of men, he had early discovered Caspar's metronomic relentlessness in the matter of beat, and had laid snares for him with Jesuitic guile. Once caged, he allowed him, as he observed to Hastings, whatever trimmings he liked; and Caspar wore his trimmings with such gusto as to endanger his pose. . . . All Tregotha tootled or sawed, and something got itself done, as the Irishman fiddled—neither by note nor ear, but by main force. Nor would Caspar stoop to the conventional programmes of village bands. The admiring tourist made note that it was the habit of Cornish fishermen to solace their leisure with Berlioz and Wagner."

Daphne, the heroine, is a daughter of the woods and sea. She is beautiful, fearless, with a frank manner, and eyes within whose depths lie thoughts as deep as the sea itself. And "mentally she was well balanced—a white soul by the interfusion of coloured rays emanating from people and things about her. At this age she recalled the pictured moods of expectancy, from Galatea to Mary; touching tiptoe the alertness of the marble and that radiant acquiescence which found signal expression in the Madonna of the Umbrian painters." We will not give away the story of her awakening. It is interesting, but disappointing, and sad to a degree. When Basil Waring, the Vicar's brother, wanders on to the rocks at Tregotha, as Daphne, viola in hand, is airing her soul—after a band practice in the school-room—on the headland under the shelter of her beloved column in the cool dusk of a summer night, and Basil slips and breaks his leg, his cry of pain arresting Daphne's music, we know pretty well what to expect. Basil Waring is the reverse of his brother the Vicar. He has tried many things, experimentally, "East Ending" among others. Miss Williams, the good-hearted shrewd spinster who watches over the well-being of the parish and seasons her remarks with kindly satire on people in general, and men in particular, thus defines gentlemanly slummers, including Basil Waring: "I know 'em—worse than stained glass, much worse. There was—let me see—there was Backhouse, down here for the autumn. He gave lectures in the school-room on Early Italian Painters, with oxy-what-you-may-call-it light; and the boys would keep treading on the pipes, and he got in a fluster—such a business as never was. So that's the sort of person he was!" To get at the atmosphere of a book one must enter into it, and *The Column* is one that must be read by readers to whom it appeals. Enough has been said to show that when this is the case no one will take it up without wishing to read to the end, for it is undoubtedly one of the cleverest works of the modern school of novels.

* "The Column." By Charles Marriott. Publisher: John Lane. Price 6s.

### For Reading to the Sick.

LOVE'S SERVICE.

To have loved once is to love God better for always. Is not human love the friend to whom God ever says, "Go up higher"? *U. A. Taylor.*

A prayer in an hour of pain,
Begun in an undertone,
Then lowered, as it would fain
Be heard by the heart alone;
A throb, when the soul is entered,
By a light that is lit above,
Where the god of nature has centered
The beauty of love—
The world is wide,—these things are small,—
They may be nothing, but they are All.

A sense of an earnest will
To help the lowly-living—
And a terrible heart-thrill,
If you have no power of giving:
An arm of aid to the weak,
A friendly hand to the friendless,
Kind words, so short to speak,
But whose echo is endless:
The world is wide—these things are small—
They may be nothing, but they are All.

*Lord Houghton.*

Our first need, whether in things spiritual or things temporal, is to be lifted out of ourselves. . . . Art thou in trouble? Others are in trouble. Art thou in bereavement? There is not a house without its vacant chair. Hast thou no thought for those who suffer what thou sufferest? Arise and look around thee. There are hearts to be bound like thine, there are tears to be dried like thine, there are days to be illumined like thine. Thy very sorrow has put a gift into thy hand—sympathy.—*G. Matheson.*

If we can reach out a helping hand to those who suffer without any mitigation, without any alleviation, depend upon it, God will give us our reward—the reward so faithfully promised by Him who said, "Whosoever shall give a cup of cold water only in the name of a disciple, verily I say unto you, he shall in no wise lose his reward.—*T. B. Dover.*

We all have our friends—thank God for that. Friendship is the very sunshine of life. It draws out all that is noblest, highest, best in a man. When we go to distant lands, our friends bid us God-speed and cheer us on our journey—just as, when the soul is taking her last journey of all, we hope to have our dear ones close at hand. Nay, who would dare to set sail across the dark river if it were not that on the other side there wait friends without number, "not lost, but gone before?"—*T. D. Dover.*

Patience and abnegation of self, and devotion to others,
This was the lesson a life of trial and sorrow had taught her.
So was her love diffused, but, like to some odorous spices,
Suffered no waste nor loss, though filling the air with aroma;
Other hope had she none, nor wish in life, but to follow
Meekly, with reverent steps, the sacred feet of her Saviour.

*Longfellow, "Evangeline."*

### Notes and Queries.

**The Editor is always willing to answer in this column, without any fee, all reasonable questions, as soon as possible.**
**But the following rules must be carefully observed:—**
1. **Every communication must be accompanied by the name and address of the writer.**
2. **The question must always bear upon nursing, directly or indirectly.**

**If an answer is required by letter a fee of half-a-crown must be enclosed with the note containing the inquiry.**

*Western Australia.*

(83) In a recent issue there was an article referring to an opening in Western Australia for Nurses. Will you kindly say where I should apply?—*Maria.*

There is only an opening for nurses who feel inclined to go out to the colony on their own account.

*Memory.*

(84) May I trouble you to give me the name of any really good system by which I could improve my memory?—*A Daughter.*

There is only one satisfactory way by which to cultivate memory. Concentrate your mind intelligently upon what you wish to retain, checking and correcting the knowledge thus gained at frequent intervals.

*Rules.*

(85) We wish to engage a maternity nurse for district work. Could you tell us where we could obtain the rules under which she should work?—*F. F.*

The Secretary of the Midwives' Institute, 12 Buckingham Street, Strand, W.C., would be able to advise you on this subject. The code of rules must be framed to suit the circumstances of the case.

*Dispensing.*

(86) Will you tell me where to write for information regarding the subjects and examinations required in order to become a dispenser?—*Nurse D.*

The Secretary of the Pharmaceutical Society, 17 Bloomsbury Square, W.C.

*Sanitary Inspector.*

(87) Could you kindly tell me what are the duties and fees of a lady sanitary inspector; where I could receive instruction; and if a nurse with two years' fever training would be eligible for the post?—*Nurse R. O.*

You will be required to pass the examination of the Sanitary Inspectors' Examination Board, 1 Adelaide Buildings, London Bridge, E.C.; or that of the Sanitary Institute, Margaret Street, W.; or of any provincial authorities under which you may work. The duties are chiefly the inspection of factories, shops, schools and dwellings; the salary £80 to £150 a year; the hours 9 A.M. to 5, or 5.30 P.M.; the holidays Saturday afternoons, Sundays, Bank Holidays, and one fortnight in the year.

*Epilepsy.*

(88) Would you kindly tell me where a man could be taken for treatment of epilepsy? His friends could afford to pay 7s. 6d. a week.—*F. C.*

The National Hospital for the Paralysed and Epileptic, Queen's Square, Bloomsbury, W.C.

*After-care Association.*

(89) Will you tell me the address of the After-care Association?—*Male Nurse.*

Church House, Dean's Yard, Westminster, S.W.

*Rules.*

(90) Will you kindly tell me the rules to be observed in sending notices of meetings of District Nursing Associations to your columns?—*Nurse H.*

You should send a copy of the report, and add any points of interest in as concise and clear a way as possible.

*Home for Domestic.*

(91) Can anyone tell me of a home in the north of England or in the Midland Counties where a middle-aged domestic servant, crippled with rheumatism, could be received free?—*Nurse F. S.*

We are afraid that the workhouse infirmary of her parish is the only place for her, unless her friends will provide for her support. If there were any hope of curing her she would be eligible for free treatment at the expense of some of the charities.

*Male Nurse.*

(92) I wrote to the manager, enclosing stamped envelope for reply, on March 8th as to training for male nurses. My letter was not for publication, and, as I have received no reply, I am afraid I ought to have addressed it to you.—*H.B.B.*

Will H.B.B. read our rules to correspondents? He will then understand why he did not receive a private reply. His question was answered April 20.

*Weston-super-Mare.*

(93) Will you kindly give me the address of the convalescent home at Weston-super-Mare where nurses are received at reduced fees? It was mentioned in the MIRROR about a year ago.—*Nurse E.*

Possibly the Royal West of England Sanatorium, Weston-super-Mare.

**Standard Books of Reference.**

"The Nursing Profession: How and Where to Train." 2s. net; post free 2s. 4d.
"Burdett's Official Nursing Directory." 3s. net.; post free, 3s. 4d.
"Burdett's Hospitals and Charities." 5s.
"The Nurses' Dictionary of Medical Terms." 2s.
"Burdett's Series of Nursing Text-Books." 1s. each.
"A Handbook for Nurses." (Illustrated.) 5s.
"Nursing: Its Theory and Practice." New Edition. 3s. 6d.
"Helps in Sickness and to Health." Fifteenth Thousand. 5s.
"The Physiological Feeding of Infants." 1s.
"The Physiological Nursery Chart." 1s.; post free, 1s. 3d.
"Hospital Expenditure: The Commissariat." 2s. 6d.

All these are published by THE SCIENTIFIC PRESS, Ltd., and may be obtained through any bookseller or direct from the publishers, 28 and 29 Southampton Street, London, W.C.

### Travel Notes.

By Our Travelling Correspondent.

### LXXII.—SOME LITTLE KNOWN PLACES IN ROME.

Those who are admirers of Hawthorn will remember his description of Hilda's Tower with the Virgin's shrine and the pigeons circling around it. It is in the Via S. Antonio dei Portoghesi, and is one of the finest mediæval towers in Rome. It is called the Torre della Scimia, and is the scene of a curious legend which one is bound to admit is met with in other countries and centuries. The story is, that once a man lived here with a favourite monkey; the creature in one of those unaccountable fits of mischief which distinguish their race, descended from his home, seized a young baby from the street below, and made his way to the summit of the tower, where he was seen by the horror-struck crowd below to be seated on the edge of the battlements, dandling the baby to and fro. The parents witnessing this appalling scene, made a vow that if the child escaped death they would burn a lamp for ever before a shrine of the Blessed Virgin, which they would erect. Presently the monkey, still grasping the baby, scrambled down as only monkeys can, and laid the child at its mother's feet. Certain it is that some curious facts in the past have called the shrine into existence and the perpetual burning of the lamp which is never extinguished either night or day. Hawthorne evidently considers the story worthy of credence.

"This palace was distinguished by a feature not very common in the architecture of Roman edifices, that is to say, a mediæval tower, square, massive, lofty, and battlemented and machicolated at the summit. At one of the angles of the battlements stood a shrine of the Virgin, such as we see everywhere at the street corners of Rome, but seldom or never, except in this solitary instance, at a height above the ordinary level of men's views and aspirations. Connected with this old tower and its lofty shrine there is a legend which we cannot pause to tell here; but for centuries a lamp has been burning before the Virgin's image, at noon, at midnight, and at all hours of the twenty-four, and must be kept burning for ever, as long as the tower shall stand, or else the tower itself, the palace, and whatever estate belongs to it, shall pass from its hereditary possessor, in accordance with an ancient vow, and become the property of the church . . . . She looked upward and saw, not indeed the flame of the never-dying lamp, which was swallowed up in the broad sunlight that brightened the shrine, but a flock of white doves, skimming, fluttering, and wheeling about the topmost height of the tower, their silver wings flashing in the pure transparency of the air."—Transformation.

This tower makes one of the most charming and picturesque corners in Rome, which indeed since the march of so-called municipal improvement have been swiftly disappearing.

### The Convent of the Sepolte Vive.

Sepolte vive means "buried alive," and round this convent which belonged to the Farnesiani nuns, much romantic conjecture hovered, and some writers have set down as solemn facts absurd prejudiced opinions for which there was absolutely no foundation. The convent existed up to 1885, and though now suppressed, it is interesting to see its site and to muse on the strange bent of mind that would willingly immure itself from the outside world as the Farnesiani nuns elected to do, for I conclude it is hardly necessary for me to impress upon the enlightened reader, the fact that retirement to this living death was an absolutely voluntary thing, and that wealthy ladies were not, as fanatic Protestants would have us believe, inveigled there by the Jesuits, and their fortunes secured by those astute gentlemen.

I first read of this convent in a novel of Buchanan's, and naturally thought it was entirely fiction, but since I have known Rome well I have learnt much about it. The entrance, carefully concealed, was close to S. Maria in Monti. Hare gives an excellent account of it:

"A narrow alley, which appeared to be a *cul de sac* ending in a picture of the Crucifixion, was in reality the approach to the Convent of the Farnesiani nuns. . . . No more curious convent has been recently destroyed. The only means of communicating with the nuns was by rapping on a barrel which projected from a wall on a platform above the roofs of the houses, when a muffled voice was heard from the interior; and if the references of the visitor were satisfactory, the barrel turned round and disclosed a key by which the initiated could admit themselves to a small chamber in the interior of the convent. Over the door was an inscription bidding those who entered that chamber to leave all worldly thought behind them. . . . In one of the walls was an opening with a double grille, beyond which was a metal plate, pierced with holes like the rose of a watering-pot. It was beyond this grille and behind this plate that the Abbess received visitors, but she was even then veiled from head to foot in heavy folds of thick serge. . . . The nuns of the Sepolte Vive are never seen again after they once assume the black veil, though they are allowed double the ordinary novitiate. They never hear anything of the outer world, even of the deaths of their nearest relations. Daily they dig their own graves and lie down in them, and their remaining hours are occupied in perpetual adoration of the Blessed Sacrament."

Mr. Hare speaks as if this order of nuns was still in existence somewhere, but so far I have not been able to learn where.

### The Protestant Cemeteries of Rome.

Perhaps it is hardly correct to speak of these as little-known places, but it is certainly true that many strangers visit the city without once going to the beautiful spot occupied by these two burial grounds, or thinking for a moment of some of the interesting personalities which sleep their last sleep in this violet-laden ground. In the older and no longer used, lies the unhappy Keats. He died in poverty and depression in a house close to the steps of Trinita de'Monti. His nature was but ill-suited to struggle in the battle of life—he desired these words to be placed on his grave: "Here lies one whose name is writ in water." Shelley wrote of this spot little thinking that his heart would once "lie close by": "The cemetery is an open space among the ruins, covered in winter with violets and daisies—it might make one in love with death to think that one should be buried in so sweet a place." After his body was thrown up by the sea at Lerice it was burned on the beach, except the heart, which was brought to Rome and placed in the new cemetery. His faithful friend, Mr. Trelawny, lies near also, whilst not far off sleeps J. A. Symonds, the gifted and charming writer of so much on Italy.

Next winter I shall hope to continue these papers on Rome; but at this season I think my readers will prefer to hear of places for summer sojourn.

### TRAVEL NOTES AND QUERIES.

Normandy, Brittany, or Belgium (Nurse Mary).—You have plenty of time before you, so that we shall be able to arrange something. Write to me again, saying how long your holiday will last, and especially the limit that you must put on your expenses. Also tell me what your tastes are, whether you prefer scenery or old world towns, inland or sea coast, and any other particulars you like, and I will give you some alternative tours. It is unfortunate that you are so far North, but there are very cheap tourist tickets which effect a great saving in the summer and autumn.

Pau and the Mountains (Semi-invalid).—I think Pau is delightful up to the end of May, and then it would be well for you to seek the mountains. You must consult your doctor as to the precise spot, because even if you do not take the baths, the climate varies a good deal. Eaux-bonnes and Eaux-Chaudes are both lovely spots, so is Cauterets, so is Luchon. If as I gather you can get about a good deal, these places offer great attractions, the walks and drives are perfect. You might return to Pau in the early part of October or even September, should it turn cold and wet in the mountains.

Knokke in Belgium (Scala).—It is a very small, flat, and ugly place near to Ostend, but the living is very cheap and communication with many interesting towns is quite easy by tram and train. You can also enjoy all the gaieties of Ostend if your tastes lie that way, without incurring the heavy hotel charges of that town. You must, however, understand that in Knokke itself there are no attractions beyond fine air on the downs.

The Loire with a Bicycle (Fusilier).—It would be very enjoyable and would not cost more than about £8 for the fortnight, if you cycle entirely beyond Orleans. The chief places of interest from Orleans to Angers are Chambord, Blois, Chaumont, Amboise, Chenonceaux, Tours (from which visit Plessis lès Tours and Loches), Langeais, Azay-le-rideau-Chinon, and Saumur. The distances are not long and the roads perfect for cycling. You can send on your superfluous luggage by train at small expense. I find it a good plan to take a third-class ticket as if one was going, and then reclaim the luggage at its destination. It often avoids delay.

